# Disentangling Multiscale Cation–Anion Dynamics in Li_4_(BH_4_)(NH_2_)_3_ Using Quasielastic Neutron Scattering

**DOI:** 10.1002/smsc.70408

**Published:** 2026-09-16

**Authors:** Mohsin Abbas, Fahim Karimi, Nicolas De Souza, Dehong Yu, Peter Fouquet, Thomas Klassen, Claudio Pistidda

**Affiliations:** ^1^ Institute of Hydrogen Technology Helmholtz‐Zentrum Hereon Geesthacht Germany; ^2^ Australian Centre for Neutron Scattering ANSTO Lucas Heights New South Wales Australia; ^3^ Institut Laue‐Langevin Grenoble France; ^4^ Helmut Schmidt University University of the Federal Armed Forces Hamburg Hamburg Germany

**Keywords:** cation–anion dynamics, ionic conductivity, neutron spin‐echo, quasielastic neutron scattering, solid‐state ionic conductors

## Abstract

Elucidating the interplay between anion dynamics and cation transport is essential for the rational design of solid‐state ionic conductors. Here, we investigate the relationship between local anion motion and long‐range ${\mathrm{Li}}^{+}$ transport in ${\mathrm{Li}}_{4}({\mathrm{BH}}_{4}){\left({\mathrm{NH}}_{2}\right)}_{3}$ by combining quasielastic neutron scattering (QENS) and neutron spin‐echo (NSE) spectroscopy with complementary thermal, structural, and electrochemical investigations. Time‐of‐flight QENS analysis reveals ultrafast reorientational motion of ${\left[{\mathrm{BH}}_{4}\right]}^{-}$ units within the crystalline lattice, characterized by a low rotational activation barrier of ∼0.16 eV. This barrier is substantially lower than the ${\mathrm{Li}}^{+}$ transport activation energy (∼0.26 eV), demonstrating that thermally activated ${\left[{\mathrm{BH}}_{4}\right]}^{-}$ reorientation provides a dynamic pathway that promotes ${\mathrm{Li}}^{+}$ hopping through the lattice. In contrast, ${\left[{\mathrm{NH}}_{2}\right]}^{-}$ anions exhibit limited quasielastic broadening, indicating more restricted dynamics. Upon melting, the emergence of *Q*‐dependent quasielastic broadening reveals translational diffusion of ${\left[{\mathrm{BH}}_{4}\right]}^{-}$ species, yielding a diffusion coefficient of ∼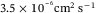. This value closely agrees with the ${\mathrm{Li}}^{+}$ diffusion coefficient obtained from ionic conductivity measurements, evidencing a transition from rotation‐assisted ${\mathrm{Li}}^{+}$ migration in the solid state to coupled cation–anion diffusion in the molten phase. Complementary NSE measurements directly capture long‐range ${\mathrm{Li}}^{+}$ motion on nanosecond timescales, providing a unified molecular‐level picture of how anion dynamics regulate ion transport in mixed–anion complex hydrides.

## Graphical abstract

Quasielastic neutron scattering disentangles BH_4_^−^ and NH_2_^−^ reorientations from Li^+^ diffusion through the solid framework and captures long‐range BH_4_^−^ translation upon melting. These findings establish a unified molecular picture of localized and diffusive motions, clarifying the dynamic landscape of ionic transport in Li_4_(BH_4_)(NH_2_)_3_.

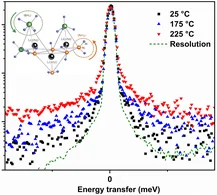

## 1 Introduction

The growing global energy demand and increasing environmental concerns have intensified the need for efficient utilization of renewable energy sources such as wind and solar power [1]. However, their inherent intermittency and the limited load flexibility of existing power grids necessitate the development of reliable off‐grid energy storage technologies [2]. In this context, advanced energy storage materials with high capacity, high energy density, and robust safety are of central importance. Metal hydrides have emerged as a particularly promising class of materials owing to their unique physicochemical properties arising from strong metal–hydrogen interactions [3]. They have been extensively studied for solid‐state hydrogen storage applications because of their high gravimetric hydrogen storage capacities, high volumetric energy densities, and due to their reversible nature of releasing and absorbing hydrogen with high degree of safety [4, 5, 6, 7]. Metal hydrides are also increasingly recognized for their multifunctionality in various fields of application, including electrochemical energy storage, thermal management, ion conductors, and compressors [8, 9, 10, 11]. A breakthrough was the discovery of fast ${\mathrm{Li}}^{+}$ ionic conductivity in ${\text{LiBH}}_{4}$ by Shin‐Ichi Orimo and coworkers in 2007, with reported conductivities on the order of ∼$10^{-3}\;S\;{\mathrm{cm}}^{-1}$ at 120 °C [12]. This finding established complex hydrides as a new class of solid‐state ionic conductors. Subsequent studies have expanded this family of ionic conductors to a wide range of compounds through cation substitution (e.g., ${\mathrm{Li}}^{+},\;{\mathrm{Na}}^{+},\;{\mathrm{Mg}}^{+2}$) and the incorporation of diverse complex anions such as ${\left[{\mathrm{BH}}_{4}\right]}^{-}$, ${\left[{\mathrm{NH}}_{2}\right]}^{-}$, ${\left[B_{10}H_{10}\right]}^{-2}$, and ${\left[{\mathrm{CB}}_{11}H_{12}\right]}^{-}$ [13, 14]. These systems exhibit highly dynamic lattices that can support fast ion transport while retaining favorable hydrogen storage characteristics, making them compelling candidates for next‐generation energy storage technologies.

Among these systems, the mixed borohydride‐amide compounds such as ${\mathrm{Li}}_{2}({\mathrm{BH}}_{4})({\mathrm{NH}}_{2})$ and ${\mathrm{Li}}_{4}({\mathrm{BH}}_{4}){\left({\mathrm{NH}}_{2}\right)}_{3}$ exhibit lithium‐ion conductivities approaching ∼$10^{-4}\;S\;{\mathrm{cm}}^{-1}$ near room temperature, significantly higher than those of the individual hydrides [15]. The coexistence of ${\left[{\mathrm{BH}}_{4}\right]}^{-}$ and ${\left[{\mathrm{NH}}_{2}\right]}^{-}$ anions introduces a complex dynamic environment that is believed to influence ${\mathrm{Li}}^{+}$ transport pathways. However, the atomic‐ and molecular‐scale origins of this enhanced conductivity remain poorly understood. In many complex hydrides, fast reorientational motion of polyanions has been proposed to facilitate lithium diffusion through the so‐called paddle‐wheel mechanism, where rotating anions transiently widen the diffusion bottlenecks between neighboring Li sites and reduce the migration energy barrier [16]. Although this concept has been widely applied to describe ion transport in complex hydrides, the extent to which anion reorientations directly govern ${\mathrm{Li}}^{+}$ migration remains an active subject of debate. Several experimental and computational studies have questioned whether rapid anion reorientations alone are sufficient to enhance ${\mathrm{Li}}^{+}$ transport, emphasizing instead the importance of correlated anion–cation motion [17, 18]. In mixed–anion systems, where multiple dynamically active species coexist, these correlations are expected to be even more complex. A central unresolved question is therefore how the rotational dynamics of ${\left[{\mathrm{BH}}_{4}\right]}^{-}$ and ${\left[{\mathrm{NH}}_{2}\right]}^{-}$ anions are temporally and spatially correlated with the translational diffusion of ${\mathrm{Li}}^{+}$, and how each contributes to the overall ionic conductivity. Critically, these processes occur over distinct timescales, ranging from picoseconds to nanoseconds, and across multiple length scales, from local molecular reorientations to long‐range ionic diffusion, making it experimentally challenging to disentangle their individual dynamical contributions.

Quasielastic neutron scattering (QENS) provides a powerful experimental approach for selectively probing the dynamics of specific atomic and molecular species through isotopic labeling, enabling their individual dynamical contributions to be separated [19]. Neutron spin‐echo (NSE) spectroscopy further extends the accessible time window to directly probe long‐range diffusive dynamics on the nanosecond timescale. The combination of these complementary neutron techniques bridges multiple spatial and temporal regimes, allowing distinct dynamical processes within multicomponent systems to be experimentally resolved. In this work, we combine TOF‐QENS, backscattering QENS, and NSE spectroscopy to disentangle the dynamics of ${\mathrm{Li}}^{+}$, ${\left[{\mathrm{BH}}_{4}\right]}^{-}$, and ${\left[{\mathrm{NH}}_{2}\right]}^{-}$ in ${\mathrm{Li}}_{4}({\mathrm{BH}}_{4}){\left({\mathrm{NH}}_{2}\right)}_{3}$. Unlike previous studies that inferred transport mechanisms primarily from macroscopic measurements or simulations, our approach experimentally resolves the individual cation and anion dynamics across multiple timescales. The combined measurements provide insight into the interplay between anion reorientational dynamics and ${\mathrm{Li}}^{+}$ transport, revealing a transition from rotation‐assisted ${\mathrm{Li}}^{+}$ hopping in the crystalline phase to coupled cation–anion diffusion in the molten state.

## 2 Results and Discussion

**FIGURE 1:**
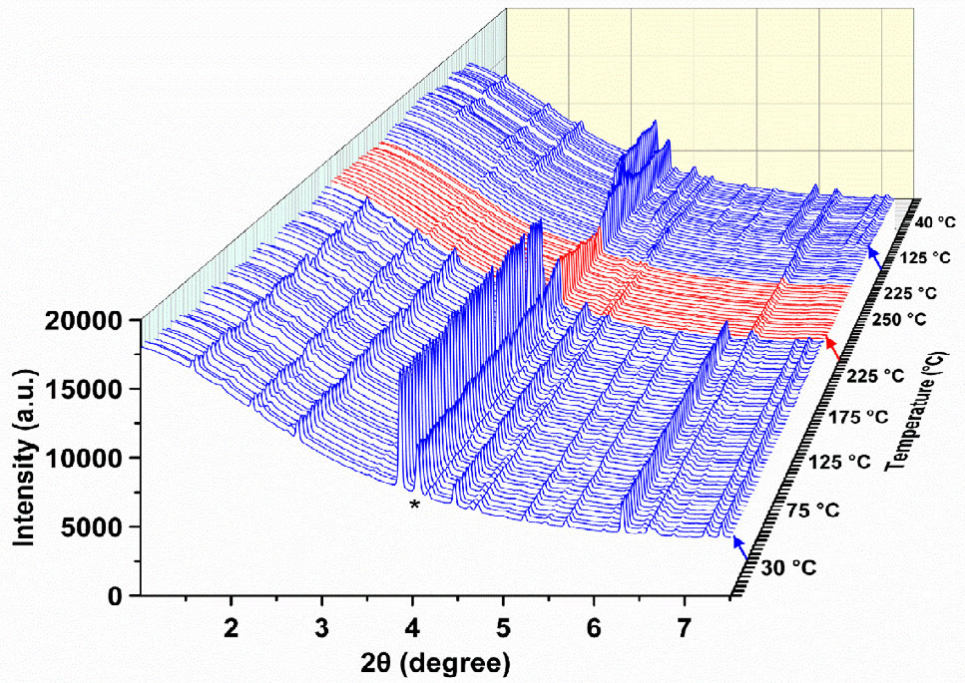
In situ XRD patterns of Li_4_(BH_4_)(NH_2_)_3_ recorded during heating from 25 to 250 °C and subsequent cooling, with heating and cooling rates of 5 and 10 °C min^−1^, respectively. The red shaded region highlights the molten state.

The thermal behavior and structural evolution of ${\mathrm{Li}}_{4}({\mathrm{BH}}_{4}){\left({\mathrm{NH}}_{2}\right)}_{3}$ were investigated through a combination of differential scanning calorimetry (DSC) and in situ XRD. The DSC analysis (Figure S1) reveals a pronounced endothermic peak with an onset ∼220 °C, corresponding to the melting of the mixed–anion phase. Upon cooling, a well‐defined exothermic peak appeared, indicating recrystallization into the thermodynamically stable ${\mathrm{Li}}_{4}({\mathrm{BH}}_{4}){\left({\mathrm{NH}}_{2}\right)}_{3}$ phase and confirming the reversibility of the thermal transition. In situ synchrotron‐radiation X‐ray powder diffraction (SR‐XPD) measurements provide direct structural insight into this thermal process. The diffraction pattern at room temperature confirms the formation of the *α*‐phase, which crystallizes in a cubic structure (space group I2_1_3), as verified by Rietveld refinement (Figure S2). As the temperature increases, the Bragg reflections gradually shift toward lower scattering angles due to thermal lattice expansion. Near ∼220 °C, the reflections characteristic of the crystalline phase start disappearing and are replaced by a broad diffuse scattering halo, indicating the loss of long‐range order and the formation of a liquid‐like state. Upon cooling, the diffraction peaks associated with the *α*‐phase reappear, demonstrating that the structural transformation is fully reversible and that the compound retains its stoichiometric integrity throughout the thermal cycle (Figure 1). Consistent with these structural observations, Fourier transform infrared (FT‐IR) spectroscopy (Figure S3) shows characteristic vibrational features of both ${\left[{\mathrm{NH}}_{2}\right]}^{-}$ and ${\left[{\mathrm{BH}}_{4}\right]}^{-}$ units, including N–H stretching modes around 3306–3244 cm^−1^ and B–H stretching modes in the range of 2363–2305 cm^−1^, together with their corresponding bending vibrations. The absence of signatures from pure ${\text{LiBH}}_{4}$ or ${\text{LiNH}}_{2}$ confirms the complete formation of the mixed–anion phase.

The ${\mathrm{Li}}^{+}$ transport properties of ${\mathrm{Li}}_{4}({\mathrm{BH}}_{4}){\left({\mathrm{NH}}_{2}\right)}_{3}$ were evaluated by electrochemical impedance spectroscopy (EIS). Owing to the mechanically soft nature of complex hydrides, the measured ionic conductivity exhibits a pronounced dependence on the pellet fabrication pressure, which strongly influences particle packing and grain‐boundary resistance [20]. Nyquist plots obtained under these conditions show a clear pressure‐dependent impedance response (Figure S4). Increasing the fabrication pressure progressively reduces the diameter of the high‐frequency semicircle, indicating improvement in particle packing density and reduces interparticle voids, which decreases the particle–particle boundary resistance. At the same time, improved mechanical contact between the pellet and the blocking electrodes helps to reduce the interfacial contribution to the measured resistance [21]. Pellets fabricated at 70–75 kN display the smallest semicircle and the most stable impedance response, reflecting the formation of optimal ion‐transport pathways. Correspondingly, the ionic conductivity measured at room temperature increases from $\;4.37\times 10^{-5}\;S\;{\mathrm{cm}}^{-1}$ for the pellet pressed at 10 kN to $2.18\times 10^{-4}\;S\;{\mathrm{cm}}^{-1}$ for the pellet fabricated at 75 kN. Under these optimized conditions, temperature‐dependent impedance measurements reveal a further increase in conductivity, reaching $1.35\times 10^{-3}\;S\;{\mathrm{cm}}^{-1}$ at 100 °C, as shown in Figure S5. These values are comparable to those reported for other highly conductive borohydride‐amide electrolytes, confirming that ${\mathrm{Li}}_{4}({\mathrm{BH}}_{4}){\left({\mathrm{NH}}_{2}\right)}_{3}$ belongs to the emerging class of fast lithium‐ion conductors. The systematic temperature dependence of the conductivity further enables extraction of the activation energy for ${\mathrm{Li}}^{+}$ migration through Arrhenius analysis, providing quantitative insight into the thermally activated transport process in this mixed–anion framework. To gain a deeper insight into the ion‐transport mechanism, the temperature‐dependent conductivity data were analyzed using the Arrhenius Equation:

(1) $$\sigma =\sigma_{0}\;\times \exp\;(-E_{a}/k_{B}T)$$

where σ is the ionic conductivity, $\sigma_{0}$ is the pre‐exponential factor, $E_{a}$ the is activation energy, $k_{B}$ the is Boltzmann constant and T is the absolute temperature. The resulting plot of ln(σ) versus 1000/T (Figure S6) exhibits a clear linear trend, indicating that lithium‐ion transport in ${\mathrm{Li}}_{4}({\mathrm{BH}}_{4}){\left({\mathrm{NH}}_{2}\right)}_{3}$ proceeds via a thermally activated hopping process. From the slope of the linear fit, an activation energy of 0.26 eV was obtained, which is relatively low and consistent with facile ${\mathrm{Li}}^{+}$ migration through the lattice. This low energy barrier explains the pronounced increase in ionic conductivity at elevated temperatures and supports the idea that the mixed–anion framework provides efficient conduction pathways with minimal resistance.

To further quantify the lithium‐ion mobility, the ionic conductivity of ${\mathrm{Li}}_{4}({\mathrm{BH}}_{4}){\left({\mathrm{NH}}_{2}\right)}_{3}$ was used to calculate the conductivity‐derived ${\mathrm{Li}}^{+}$ diffusion coefficient using the Nernst–Einstein Equation [22]:

(2) $$D=\frac{\sigma k_{B}T}{ne^{2}}$$

where D represents the estimated ${\mathrm{Li}}^{+}$ diffusion coefficient, σ is the measured ionic conductivity, $k_{B}$ is the Boltzmann constant ($1.381\times 10^{-23}{\mathrm{JK}}^{-1}$), T is the absolute temperature (K), n is the number density of mobile ${\mathrm{Li}}^{+}$ ions, and e is the elementary charge ($1.602\times 10^{-19}C$). The Nernst–Einstein relation assumes independent ${\mathrm{Li}}^{+}$ ion migration; therefore, the calculated conductivity‐derived diffusion coefficient represents an approximation and may differ from tracer or self‐diffusion coefficients obtained by direct diffusion measurements owing to ion correlation effects.

For ${\mathrm{Li}}_{4}({\mathrm{BH}}_{4}){\left({\mathrm{NH}}_{2}\right)}_{3}$, each formula unit contains four Li^+^ ions and assumes all four units contribute as mobile carriers. Molar mass of the compound is M=90.68 g/mol and the density is $\rho =0.82\;g/{\text{cm}}^{3}$, $N_{A}$ is Avogadro's number ($6.022\times 10^{23}{\text{ mol}}^{-1}$), the number density of ${\mathrm{Li}}^{+}$ ions can be calculated using




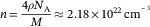




By substituting the calculated ${\mathrm{Li}}^{+}$ number density into the Nernst–Einstein Equation, the conductivity‐derived ${\mathrm{Li}}^{+}$ diffusion coefficient was estimated to be: $D\approx 1.60\times 10^{-9}\;{\mathrm{cm}}^{2}s^{-1}$ at 25 °C, increasing to $D\approx 1.24\times 10^{-8}\;{\mathrm{cm}}^{2}s^{-1}$ at 100 °C. These values are consistent with the thermally activated ionic transport inferred from the Arrhenius analysis, demonstrating that ${\mathrm{Li}}^{+}$ ions migrate efficiently through the mixed–anion framework and providing a quantitative link between macroscopic conductivity and microscopic ion motion. Remarkably, near the melting point, the system exhibits a much higher diffusion coefficient of $D\approx 2.1\times 10^{-6}\;{\mathrm{cm}}^{2}s^{-1}$ [23], reflecting the enhanced mobility associated with the liquid‐like state observed in the DSC and in situ XRD measurements.

While impedance spectroscopy provides access to macroscopic ionic transport and the Nernst–Einstein analysis yields an estimate of the ${\mathrm{Li}}^{+}$ diffusion coefficient, these approaches do not directly reveal the microscopic motions that facilitate ion migration within the mixed–anion lattice. In complex hydrides, the rotational dynamics of molecular anions are often strongly coupled with cation diffusion, and identifying these motions is therefore essential for understanding the origin of the high ionic conductivity observed in ${\mathrm{Li}}_{4}({\mathrm{BH}}_{4}){\left({\mathrm{NH}}_{2}\right)}_{3}$. To probe these local dynamics directly, QENS measurements were performed using a time‐of‐flight (TOF) spectrometer, which is ideally suited for detecting stochastic motions on the subnanosecond (≈0.1–100 ps) timescale [24]. Because hydrogen possesses an exceptionally large incoherent neutron scattering cross section, QENS is particularly sensitive to the dynamics of hydrogen‐containing species. To selectively probe the dynamics of the ${\left[{\mathrm{BH}}_{4}\right]}^{-}$ anions while suppressing contributions from ${\left[{\mathrm{NH}}_{2}\right]}^{-}$ groups, a partially deuterated sample ^7^Li_4_(^11^BH_4_)(ND_2_)_3_ was synthesized (Figures S9 and S10). The use of the ^11^B isotope minimizes neutron absorption associated with normal boron, while substitution of H by D in ${\left[{\mathrm{NH}}_{2}\right]}^{-}$ or in ${\left[{\mathrm{BH}}_{4}\right]}^{-}$ groups effectively reduces the huge incoherent scattering cross‐section of H (≈ 80 barns) due to the significantly smaller incoherent scattering cross‐section of deuterium D (≈ 2 barns). Hence, this isotopic labeling strategy allows us to selectively measure the dynamic signal of individual anion groups by applying QENS.

**FIGURE 2:**
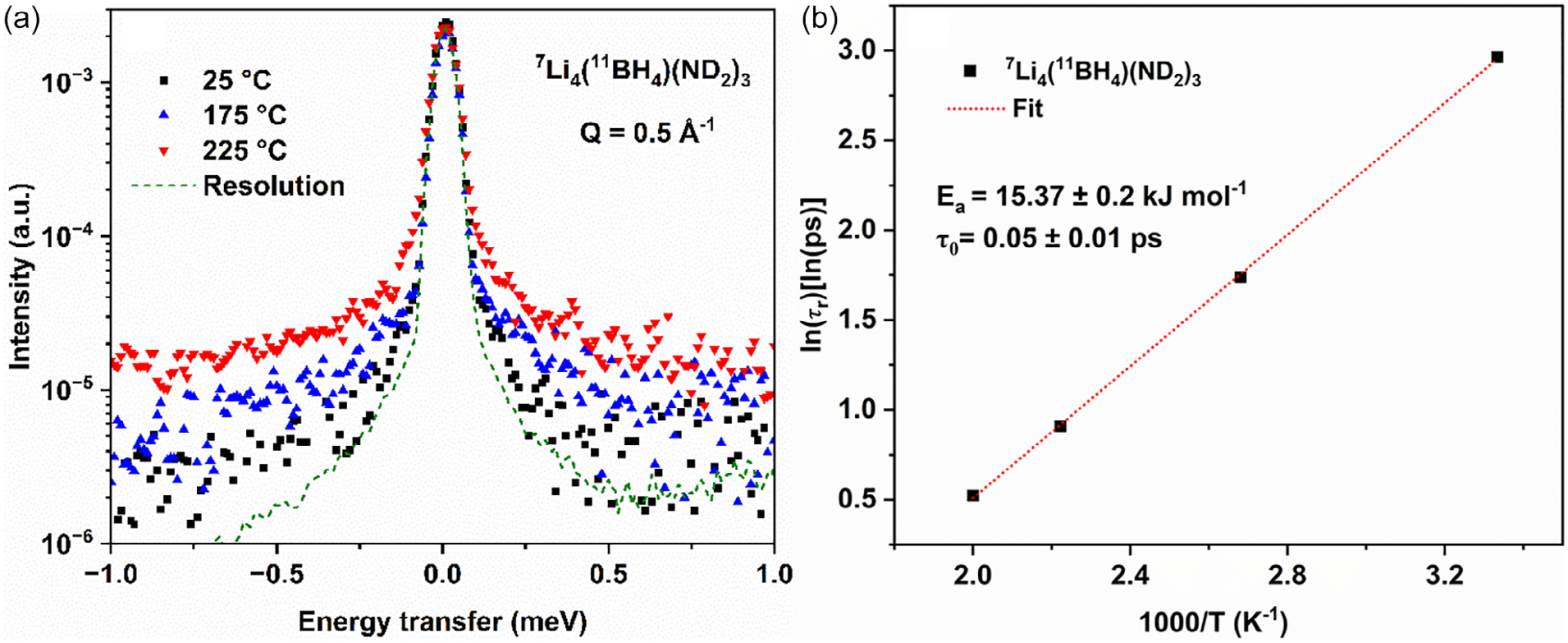
(a) QENS spectra of ^7^Li_4_(^11^BH_4_)(ND_2_)_3_ measured at 25, 175, and 225 °C along with the instrumental resolution function, recorded using a time‐of‐flight (TOF) spectrometer. (b) Arrhenius plot of the rotational residence times used to determine the activation energy barrier for BH_4_^−^ reorientational motion.

Figure 2a shows the dynamic structure factor S(Q,ω) of ^7^Li_4_(^11^BH_4_)(ND_2_)_3_ measured at 25, 175, and 225 °C together with the instrumental resolution obtained from a vanadium standard. Notably, quasielastic broadening is already present at room temperature, suggesting that the rotational motion of the borohydride units occurs well below the temperature range where macroscopic lithium conductivity becomes significant. With increasing temperature, a pronounced broadening of the elastic line is observed, indicating the onset of rapid stochastic motions of the ${\left[{\mathrm{BH}}_{4}\right]}^{-}$ tetrahedra. To quantify the dynamics in this temperature range, the QENS spectra were fitted by using the model shown in Equation (3). There, the delta function (*δ*) is included to describe the elastic component of the scattering, a Lorentzian function (*L*) is added for the quasielastic scattering contribution of the sample, and a flat background (bg) is assumed to account for several effects that create a background such as multiple scattering or dynamic of higher order such as phonons, etc. The model function was convolved with the instrumental resolution function (*R*), determined from a vanadium standard.

(3) $$S\left(Q,\omega \right)=R⊗[A_{0}(Q)\delta (\omega )+A_{1}(Q)L(\omega ,\Gamma )]+bg$$

where $A_{0}(Q)$ and $A_{1}(Q)$ represent the elastic and quasielastic amplitudes, respectively, Γ represents the HWHM of a Lorentzian function. The Lorentzian linewidth is directly related to the relaxation rate of the underlying motion of the observed species, Γ=ℏ/τ, where ℏ is the reduced Planck constant, τ is the characteristic relaxation time of the species. The model fits the experimental data measured at 100 °C and is shown in Figure S11.

The *Q*‐dependence of the FWHM at different temperatures shows only a weak variation over the measured *Q* range, indicating that the dynamics are largely *Q*‐independent (Figure S12). Within QENS theory, a Q‐independent linewidth is characteristic of localized motions, such as rotational or confined dynamics, whereas a finite Q‐dependence is indicative of long‐range translational diffusion [19]. The observed behavior therefore points to rapid reorientations of the ${\left[{\mathrm{BH}}_{4}\right]}^{-}$ tetrahedra around C3 axis.

The temperature dependence of the linewidths was further analyzed using the Arrhenius relation:

(4) $$\tau_{r}=\tau_{0}\exp\left(\frac{E_{a}}{K_{B}T}\right)$$

where $\tau_{r}$ is the mean residence time for each successive rotational step derived from the Lorentzian FWHM, τ∼2ℏ/FWHM. An activation barrier for ${\left[{\mathrm{BH}}_{4}\right]}^{-}$ reorientation of approximately $E_{a}\approx 15.5\text{ kJ }{\mathrm{mol}}^{-1}\approx 0.16\;\mathrm{ev}$ was obtained through an Arrhenius plot (Figure 2b). This value is substantially lower than the activation energy associated with ${\mathrm{Li}}^{+}$ transport (∼0.26 eV), indicating that ${\left[{\mathrm{BH}}_{4}\right]}^{-}$ reorientational motion occurs significantly faster than ${\mathrm{Li}}^{+}$ migration. The high mobility of the borohydride units introduces additional structural flexibility within the mixed–anion framework, creating a dynamic lattice environment that facilitates ${\mathrm{Li}}^{+}$ migration and supports enhanced ionic conductivity. Such rapid anion reorientations are consistent with a paddle‐wheel‐type mechanism, where the reorientational dynamics of anions promote cation transport by providing a structurally dynamic framework and reducing constraints for cation migration within the mixed–anion lattice [25, 26, 27]. This interpretation is further supported by previous studies showing that replacing “slower” rotating $PS_{4}^{3-}$ anions with the “faster” paddle‐wheel $\mathrm{Si}S_{4}^{4-}$ leads to enhanced lithium‐ion transport, highlighting the important role of anion rotational dynamics in promoting ionic conductivity [28].

Tsai et al. performed NMR measurements and AIMD simulations for ${\mathrm{Na}}_{3-x}O_{1-x}{\left({\mathrm{BH}}_{4}\right)}_{x}({\mathrm{NH}}_{2})\;(x=0-1)$, which highlight the cooperative nature of anion‐driven transport [29]. These studies show that ${\mathrm{Na}}^{+}$ diffusion is strongly coupled to the rotational mobility of both ${\left[{\mathrm{BH}}_{4}\right]}^{-}$ and ${\left[{\mathrm{NH}}_{2}\right]}^{-}$ anions, where the motion of each species contributes to enabling fast ion transport, effectively giving rise to a double paddle‐wheel mechanism involving two dynamically active anions. In the present study, QENS measurements directly resolve the ultrafast reorientational dynamics of the ${\left[{\mathrm{BH}}_{4}\right]}^{-}$ units. In contrast, the quasielastic contribution from ${\left[{\mathrm{NH}}_{2}\right]}^{-}$ remains weak within the accessible instrumental resolution, suggesting comparatively more sluggish and restricted motion. The preserved structural integrity of both anions together with the high ionic conductivity indicates that ${\left[{\mathrm{NH}}_{2}\right]}^{-}$ participates in the cooperative transport process. This interpretation is further supported by measurements performed using the EMU backscattering spectrometer (BS) (Figure S14), and our previous study [30]. Taken together, these results suggest that lithium transport in ${\mathrm{Li}}_{4}({\mathrm{BH}}_{4}){\left({\mathrm{NH}}_{2}\right)}_{3}$ is influenced by the coupled dynamics of both anionic species in the crystalline phase, consistent with a double paddle‐wheel‐type transport mechanism involving two dynamically active anions.

Upon further heating, the dynamic behavior of 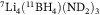 changes markedly as the system approaches the melting transition at ∼220 °C, as independently confirmed by the DSC and in situ SR‐XPD measurements discussed above. In this temperature regime, attempts to describe the QENS spectra using the localized rotational model employed for the crystalline phase were no longer successful. Instead, the quasielastic linewidth Γ shows a pronounced increase with the scattering vector Q, which is characteristic of long‐range translational motion rather than purely localized dynamics. This behavior indicates that the ${\left[{\mathrm{BH}}_{4}\right]}^{-}$ units acquire significant translational mobility in the molten state. To capture this behavior, the spectra were analyzed using a model that accounts for two simultaneous and statistically independent motions: long‐range translational diffusion and localized reorientational motion. In this framework, the total scattering function is expressed as

(5) $$S(Q,\omega )=R⊗\left[A_{0}(Q)L_{t}(Q,{\text{Γ}}_{t})+A_{1}(Q)L_{c}(Q,{\text{Γ}}_{c})\right]+bg$$

where $L_{t}$ and $L_{c}$ represent Lorentzian functions associated with translational and localized dynamics, respectively, R is the instrumental resolution function, and bg accounts for a flat background. The resulting fits (Figure S13) reveal two distinct quasielastic contributions. The linewidth associated with the translational component exhibits a clear Q‐dependent broadening, whereas the localized component remains nearly Q‐independent, consistent with fast rotational motion superimposed on translational diffusion. From these analyses, the linewidth of the localized component was determined to be $\Gamma_{c}=740\pm 70\;\text{µ}\mathrm{eV}$, corresponding to characteristic timescales of ≈0.9 ps. Compared with the crystalline phase, this result demonstrates a significant acceleration of the reorientational dynamics of the ${\left[{\mathrm{BH}}_{4}\right]}^{-}$ units in the molten state.

The Q‐dependent linewidth associated with translational motion ($\Gamma_{t}$) was further analyzed using the isotropic jump‐diffusion model proposed by Chudley and Elliott, in which the quasielastic linewidth follows Equation (6) [31].

(6) $$\text{Γ}\left(Q\right)=\frac{\hbar }{\tau }\left[1-\frac{\sin\;(Ql)}{Ql}\right]$$

where l is the characteristic jump length and τ is the residence time between successive jumps. The corresponding self‐diffusion coefficient is given by

(7) $$D=\frac{l^{2}}{6\tau }$$

**FIGURE 3:**
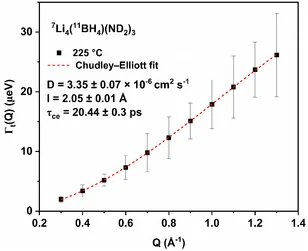
*Q*‐dependent quasielastic linewidth fitted using the Chudley–Elliott jump diffusion model to describe the translational motion in the molten phase.

Fitting the experimental data with this model yields a residence time of ≈20 ps and a jump length of 2.01 Å, resulting in a diffusion coefficient of $D\approx 3.5\times 10^{-6}{\text{ cm}}^{2}\;s^{-1}$ for the molten phase at 220 °C (Figure 3). Remarkably, this value is of the same order of magnitude as the ${\mathrm{Li}}^{+}$ diffusion coefficient (∼$2.1\times 10^{-6}\;{\text{cm}}^{2}\;s^{-1}$) estimated from ionic conductivity near the melting point, indicating that the translational mobility of the ${\left[{\mathrm{BH}}_{4}\right]}^{-}$ units becomes comparable to that of the lithium ions in the liquid‐like state. This close correspondence highlights the strongly coupled nature of cation and anion dynamics in the molten phase and further supports the notion that increasing anion mobility progressively facilitates lithium transport as the lattice approaches melting.

While the TOF‐QENS measurements described above reveal the ultrafast reorientational and translational dynamics of the ${\left[{\mathrm{BH}}_{4}\right]}^{-}$ anions on the picosecond timescale, investigating long‐range dynamics associated with lithium‐ion transport requires access to significantly longer time windows. Therefore, NSE spectroscopy was applied to probe slower ${\mathrm{Li}}^{+}$ dynamics on the nanosecond timescale. Since NSE measures the coherent intermediate scattering function of the entire sample, the contribution of all nuclei to the measured signal must be considered. The coherent neutron scattering cross‐sections of 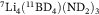 were therefore calculated and are summarized in Table S1. The ^7^Li contribution accounts for ≈2.55% of the total coherent scattering cross‐section. Thus, the interpretation of NSE relaxation requires consideration not only of the relative scattering contributions but also of the characteristic spatial and temporal signatures of the observed dynamics and their consistency with independent measurements of ion transport.

**FIGURE 4:**
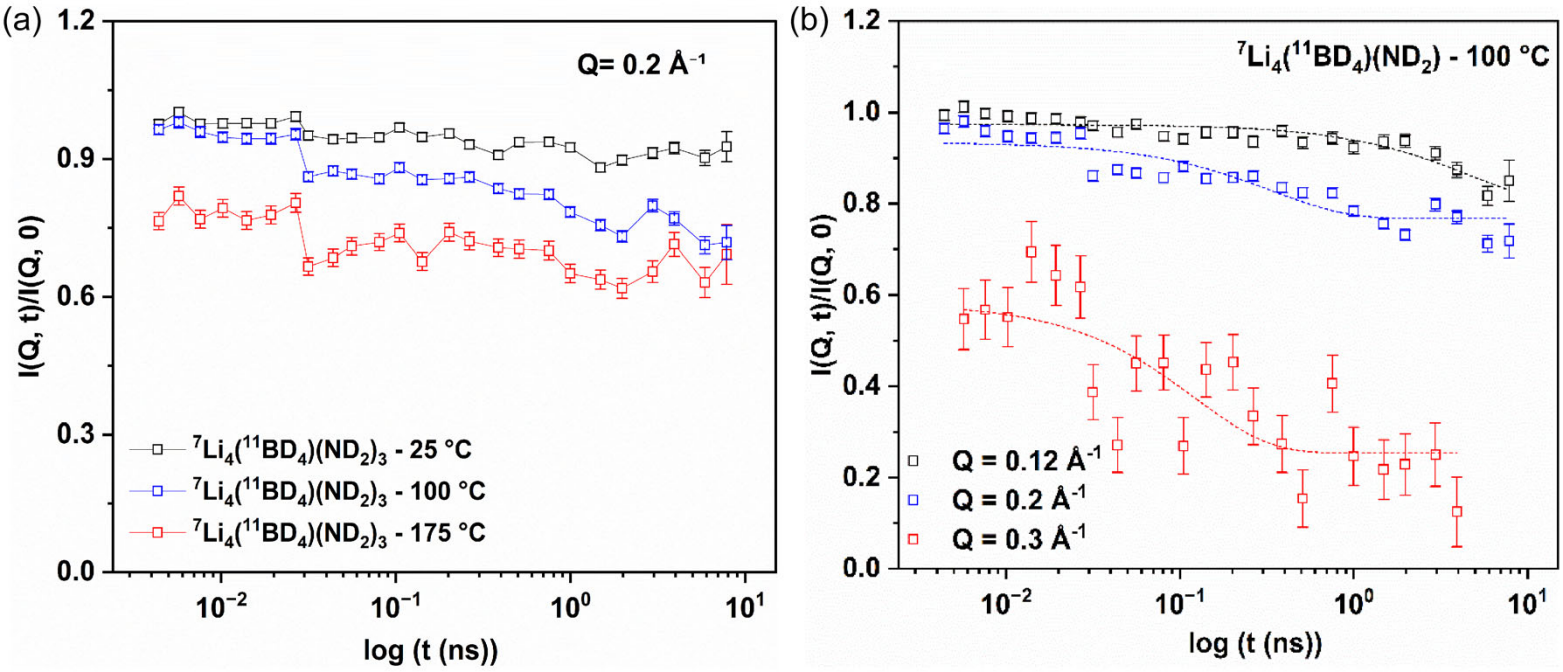
(a) Neutron spin‐echo spectra of ^7^Li_4_(^11^BD_4_)(ND_2_)_3_ measured at *Q* = 0.2 Å^−1^. (b) Single‐exponential model fits applied to the NSE data ^7^Li_4_(^11^BD_4_)(ND_2_)_3_ at *Q* = 0.12, 0.20, and 0.30 Å^−1^ measured at 100 °C.

NSE measurements were performed on the isotopically enriched compound 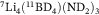 at 25, 100, and 175 °C using the WASP spectrometer at ILL, with ≈4 h of measurement time per temperature [32]. Figure 4a shows the intermediate scattering function, I(Q,t)/I(Q,0), measured at $Q=0.2\;{Å}^{-1}$. A measurable decay of the correlation function is observed within the accessible Fourier time window, indicating the presence of slow diffusive dynamics on the nanosecond timescale. The relaxation time is most pronounced at low *Q* values, whereas at larger momentum transfers the decay becomes very small and noisy, consistent with the fact that long‐range ${\mathrm{Li}}^{+}$ diffusion is primarily probed at smaller scattering vectors. The localized motions of the anionic species identified by TOF‐QENS occur on significantly faster timescales and therefore are not expected to contribute substantially within the NSE time window.

The observed relaxation was analyzed using a single‐exponential relaxation model.

$$I(Q,t)=A\;e^{-t/\tau }+C$$

where A represents the dynamic amplitude, τ is the characteristic relaxation time, and C accounts for the time‐independent elastic contribution. Fits to the NSE spectra measured at Q=0.12, 0.20, and $0.30\;{Å}^{-1}$ at 100 °C are shown in Figure 4b. The extracted relaxation times are 4.63 ns, 0.33 ns, and 0.12 ns, respectively. The systematic decrease of τ with increasing Q is characteristic of diffusive lithium‐ion motion, where larger momentum transfers correspond to shorter length scales and faster relaxation processes.

The relaxation time observed at low Q(∼4–5 ns) agrees well with the residence time estimated from the ${\mathrm{Li}}^{+}$ diffusion coefficient obtained from ionic conductivity measurements. Using the diffusion coefficient derived from the Nernst–Einstein analysis and assuming a characteristic jump length on the order of 2 Å, the corresponding residence time is estimated to lie in the range of 4–6 ns, closely matching the NSE results. Furthermore, analysis of the NSE data at $Q=0.12\;{Å}^{-1}$ and 100 °C using a jump model yields a ${\mathrm{Li}}^{+}$ diffusion coefficient of $1.44\times 10^{-8}{\text{ cm}}^{2}\;s^{-1}$, in good agreement with the independently derived ${\mathrm{Li}}^{+}$ diffusion coefficient obtained from ionic conductivity measurements using the Nernst–Einstein relation. This agreement provides strong evidence that the observed NSE relaxation corresponds to long‐range ${\mathrm{Li}}^{+}$ transport dynamics.

At room temperature, however, the diffusion coefficient derived from conductivity measurements corresponds to an estimated relaxation time of ≈40 ns, which exceeds the accessible NSE Fourier time window for this experiment. Consequently, no measurable decay of the intermediate scattering function is detected at 25 °C, whereas at 100 °C the ${\mathrm{Li}}^{+}$ motion becomes sufficiently fast to fall within the measurable time range, allowing direct observation of lithium diffusion.

Together, the TOF‐QENS, backscattering, and NSE measurements establish a multiscale picture of ion transport in ${\mathrm{Li}}_{4}({\mathrm{BH}}_{4}){\left({\mathrm{NH}}_{2}\right)}_{3}$, where the ${\left[{\mathrm{BH}}_{4}\right]}^{-}$ picosecond anion reorientational dynamics, nanosecond ${\mathrm{Li}}^{+}$ diffusion, and macroscopic conductivity measurements are directly connected across multiple time and length scales.

## 3 Conclusion

In summary, the ionic transport mechanism in ${\mathrm{Li}}_{4}({\mathrm{BH}}_{4}){\left({\mathrm{NH}}_{2}\right)}_{3}$ has been elucidated by combining structural characterization, electrochemical measurements, and QENS spectroscopy techniques spanning multiple time and length scales. Thermal analysis and in situ synchrotron X‐ray diffraction confirm that the material remains structurally stable up to ∼220 °C before undergoing a reversible transition to a molten state. EIS reveals significant lithium‐ion conductivity, reaching $1.35\times 10^{-3}\;{S\;\mathrm{cm}}^{-1}$ at 100 °C with an activation energy of 0.26 eV, corresponding to ${\mathrm{Li}}^{+}$ diffusion coefficients on the order of $10^{-9}{\text{cm}}^{2}s^{-1}$ in the crystalline phase. TOF QENS measurements show that ${\left[{\mathrm{BH}}_{4}\right]}^{-}$ tetrahedra undergo rapid reorientational motion on the picosecond timescale, with a rotational barrier of ∼0.16 eV. These ultrafast anion dynamics occur significantly faster than ${\mathrm{Li}}^{+}$ hopping and support a paddle‐wheel‐type mechanism, in which rotating anions dynamically facilitate lithium migration within the mixed–anion framework. Upon melting, the QENS spectra reveal the emergence of translational diffusion of the ${\left[{\mathrm{BH}}_{4}\right]}^{-}$ units, with a diffusion coefficient of ∼$3.5\times 10^{-6}{\text{cm}}^{2}s^{-1}$. This value closely matches the ${\mathrm{Li}}^{+}$ diffusion coefficient (∼$2\times 10^{-6}{\text{cm}}^{2}s^{-1}$) estimated from ionic conductivity near the melting point, indicating that both cations and anions participate in translational motion. Complementary NSE measurements directly probe lithium dynamics on the nanosecond timescale, yielding relaxation times consistent with the residence times derived from the conductivity‐based diffusion coefficients. Together, these results reveal a clear evolution of the transport mechanism: in the crystallinephase, rapid ${\left[{\mathrm{BH}}_{4}\right]}^{-}$ anion reorientations assist ${\mathrm{Li}}^{+}$ hopping, whereas in the molten state, the system transitions to a coupled ionic diffusion regime in which both ${\mathrm{Li}}^{+}$ and ${\left[{\mathrm{BH}}_{4}\right]}^{-}$ ions contribute to long‐range mass transport. By disentangling the dynamics of ${\mathrm{Li}}^{+}$ and ${\left[{\mathrm{BH}}_{4}\right]}^{-}$across multiple timescales, this work provides a mechanistic framework for understanding ion transport in mixed–anion complex hydrides and highlights the critical role of anion dynamics and lattice flexibility in enabling fast lithium conduction.

## 4 Experimental Section/Methods

### 4.1 Materials Preparation

**Synthesis of Li**_**4**_ **(BH**_**4**_ **)(NH**_**2**_ **)**_**3**_: Li_4_(BH_4_)(NH_2_)_3_ was synthesized by high‐energy ball milling of LiBH_4_ and LiNH_2_ in a 1:3 molar ratio using a SPEX 8000 M mixer mill inside a glovebox. Milling was performed for 45 min at a BPR of 10:1. The resulting powder was annealed at 120 °C for 12 h under ∼100 bar hydrogen pressure to remove milling‐induced defects and improve crystallinity.

**Synthesis of** ^**7**^ **LiND**_**2**_ **and** ^**7**^ **LiNH**_**2**_: ^7^LiND_2_ was synthesized through a gas–solid reaction between ^7^Li metal and deuterated ammonia (ND_3_) gas via a ball milling‐assisted method. Metallic ^7^Li was cleaned with anhydrous hexane to remove surface contaminants, cut into small pieces, and loaded into a stainless‐steel high‐pressure milling vial. The vial was sealed and pressurized with ND_3_ gas to 5 bar, maintaining a stoichiometric ratio for complete conversion. Ball milling was conducted at 100 rpm with a BPR of 10:1 using stainless steel media. The first milling protocol comprised a total of 40 cycles (6 min milling + 3 min rest per cycle; ∼4 h of total ball milling time). After the initial milling stage, the vial was recharged with fresh ND_3_ (5 bar) and subjected to an additional 10 milling cycles under identical conditions. A similar procedure was followed for the synthesis of ^7^LiNH_2_, using NH_3_ instead of ND_3_. The phase purity and successful synthesis of ^7^LiND_2_ were confirmed by PXD and FT‐IR analysis.

**Synthesis of** ^**7**^ **Li** ^**11**^ **BD**_**4**_ **:** Synthesis of ^7^Li^11^BD_4_ was performed via H–D exchange by exposing ^7^Li^11^BH_4_ to 30 bar of D_2_ gas at 260 °C using a high‐pressure reactor. First, the sample was loaded into the reactor under an argon atmosphere, and the reactor was evacuated and purged several times with D_2_ gas to ensure complete removal of argon gas. During the H–D exchange process, the D_2_ gas was refilled every 8 h to maintain a consistent deuterium atmosphere. The extent of H–D exchange was monitored by collecting samples after 3 days and subsequently at regular intervals up to 2 weeks. The successful synthesis and phase purity of ^7^Li^11^BD_4_ were confirmed by PXD and FT‐IR analysis.

**Synthesis of** ^**7**^ **Li**_**4**_ **(** ^**11**^ **BH**_**4**_ **)(ND**_**2**_ **)**_**3**_ **, Li**_**4**_ **(BD**_**4**_ **)(NH**_**2**_ **)**_**3**_ **,** **and** ^**7**^ **Li**_**4**_ **(** ^**11**^ **BD**_**4**_ **)(ND**_**2**_ **)**_**3**_ **:** ^7^Li_4_(^11^BH_4_)(ND_2_)_3_, Li_4_(BD_4_)(NH_2_)_3,_ and ^7^Li_4_(^11^BD_4_)(ND_2_)_3_ were synthesized via high‐energy ball milling of the corresponding reactants in a stoichiometric ratio (1:3), following the same procedure as described in the previous section. The only change was that the postannealing was performed under one bar of Ar. The formation and purity of the final phases were confirmed by PXD and FT‐IR.

### 4.2 Methods

**DSC:** In this work, DSC experiments were conducted using a Netzsch DSC 204 calorimeter placed inside an argon‐filled glovebox to maintain H_2_O and O_2_ levels below 1 ppm. A sample of 5–10 mg was placed in Al_2_O_3_ sample crucibles and heated from room temperature to 250 °C at a heating rate of 5 °C/min.

**FT‐IR spectroscopy**: An Agilent Technologies Cary 630 FT‐IR spectrometer was used inside an argon‐filled glovebox to maintain water (H_2_O) and oxygen (O_2_) levels below 0.1 ppm. Measurements were conducted in the transmission mode over a spectral range of 650 to 4000 cm^−1^ with a resolution of 4 cm^−1^. This setup enabled the precise detection of molecular vibrations, which provided detailed insights into the structural characteristics of our samples.

**In situ SR‐XPD:** In situ SR‐XPD measurements were performed at beamline P02.1 of PETRA III, Deutsches Elektronen‐Synchrotron (DESY), Hamburg, Germany, using X‐rays with a wavelength of 0.206 Å [33]. A custom‐designed in situ reaction cell was used for these experiments. Approximately 1–2 mg of the sample was loaded into a sapphire capillary (inner diameter: 0.8 mm; outer diameter: 1.0 mm), which was subsequently mounted on the reaction cell. The capillary was sealed at both ends using graphite ferrules to ensure an airtight environment and maintain sample integrity under hydrogen atmosphere. A thermocouple was inserted into the capillary in direct contact with the powder, which enabled precise temperature monitoring at the beam interaction point. A microheater, positioned directly below the capillary, was used to uniformly heat the sample during the measurement. This configuration ensured reliable thermal control throughout the in situ diffraction experiment. SR‐XPD data were collected every 10 s during heating and cooling, and the resulting 2D diffraction images were converted into text files using FIT2D [34]. Phase identification was confirmed by comparison with reference patterns from ICSD database.

**Ionic conductivity measurements:** The CompreDrive system (rhd instruments, Darmstadt, Germany) was used for pressure‐ and temperature‐dependent ionic conductivity studies. Powder samples (200 mg) were prepressed into pellets with a thickness of 1 mm and a diameter of 10 mm and placed inside a 12C CompreCell with a 12 mm inner diameter. A uniaxial fabrication pressure was applied for 10 min to ensure accurate measurement (±0.5%) and the heating mantle maintained a constant temperature during these measurements. Electrochemical impedance analysis was performed after achieving thermal and pressure equilibrium. Fabrication pressures of 10, 30, 50, 70, and 75 kN were tested to optimize ionic conductivity. After the optimization of fabrication pressure, temperature‐dependent ionic conductivity measurements were performed. Data analysis was carried out using RelaxIS3 software for model fitting.

The ionic conductivity (*σ*) was calculated using the standard relation:

σ=L/R.A

where *L* = 1 mm is the sample thickness, *A* = 0.785 cm^2^ is the cross‐sectional area of the pellet (10 mm diameter), and *R* is the bulk resistance obtained from impedance measurements.

**QENS: TOF spectrometer measurements:** QENS measurements on Li_4_(BH_4_)(ND_2_)_3_ were performed on the Pelican TOF spectrometer at ANSTO to analyze BH_4_^−^ dynamics [24]. Approximately 400 mg of the sample was loaded into similar cylindrical holders. Neutrons with a wavelength of 4.9 Å were used, covering a *Q* range of 0.2–2.5 Å^−1^. Data were collected at 25, 100, 175, and 225 °C, with vanadium and empty holder measurements for instrument resolution and background correction. Data analysis and data reduction were performed using Dave and Mantid, respectively [35, 36].

**QENS: BS measurements:** QENS measurements on Li_4_(BD_4_)(NH_2_)_3_ were performed using the EMU BS at ANSTO. To maximize neutron transmission, the sample was prepared as a thin single layer on aluminum foil and placed in a cylindrical holder. Measurements were conducted at 25, 100, 175, and 225 °C. Slower motions of NH_2_^−^ were probed using the EMU BS with λ = 6.28 Å, covering *Q* = 0.3–1.9 Å^−1^, with an energy resolution of 1.1 μeV. Vanadium and empty holder measurements were used for instrument resolution and background correction.

**NSE analysis:** NSE measurements were performed on ^7^Li_4_(^11^BD_4_)(ND_2_)_3_ using the WASP instrument at the Institut Laue‐Langevin (ILL), France, to analyze Li^+^ motion [32]. Samples were packed inside an argon‐filled glovebox into concentric cylindrical cells (15 mm outer diameter, 0.5 mm radial gap) and sealed with lead wire to ensure an air‐tight seal. A neutron wavelength of 6 Å was selected to access the Fourier‐time window needed for atomic and molecular relaxations on WASP. At this wavelength, the instrument provides a maximum Fourier time of about 5 ns and probes dynamics in the subnanosecond to nanosecond range. The measured *Q* range was 0.12–1.88 Å^−1^. Data were collected at 25, 100, and 175 °C, with 4 h acquisition time at each temperature. An empty cell of identical geometry was measured for background subtraction. Data were subsequently normalized to a measurement from a fully elastically scattering TiZr sample. Data reduction followed the standard ILL routines implemented in Igor Pro 8.

## Author Contributions

M. Abbas wrote the original draft and contributed to conceptualization, methodology, validation, formal analysis, and investigations. F. Karimi contributed to investigation, formal analysis, and conceptualization. N. D. Souza contributed to investigation and conceptualization. D. Yu contributed to investigation and methodology. P. Fouquet contributed to investigation, methodology, and conceptualization. T. Klassen contributed to funding acquisition and project administration. C. Pistidda contributed to supervision, conceptualization, resources, and funding acquisition.

## Conflicts of Interest

The authors declare no conflicts of interest.

## Supporting information

Supplementary Material

## Data Availability

The data supporting the findings of this study are available from the corresponding author upon request.
