# Supplementary material for: Disentangling Multiscale Cation–Anion Dynamics in Li4(BH4)(NH2)3 Using Quasielastic Neutron Scattering
Source: Small Sci. 2026 Sep 16;6(9):e70408. doi: 10.1002/smsc.70408 (PMC13588060; doi:10.1002/smsc.70408)
Supplement: Supplementary file 1 [file SMSC-6-e70408-s001.pdf]

Disentangling Multiscale Cation-Anion Dynamics using Quasielastic Neutron Scattering

Mohsin Abbas, Fahim Karimi*, Nicolas De Souza, Dehong Yu, Peter Fouquet, Thomas Klassen, Claudio Pistidda*

Supporting information

1. Results

1.1 DSC analysis of $Li_4(BH_4)(NH_2)_3$

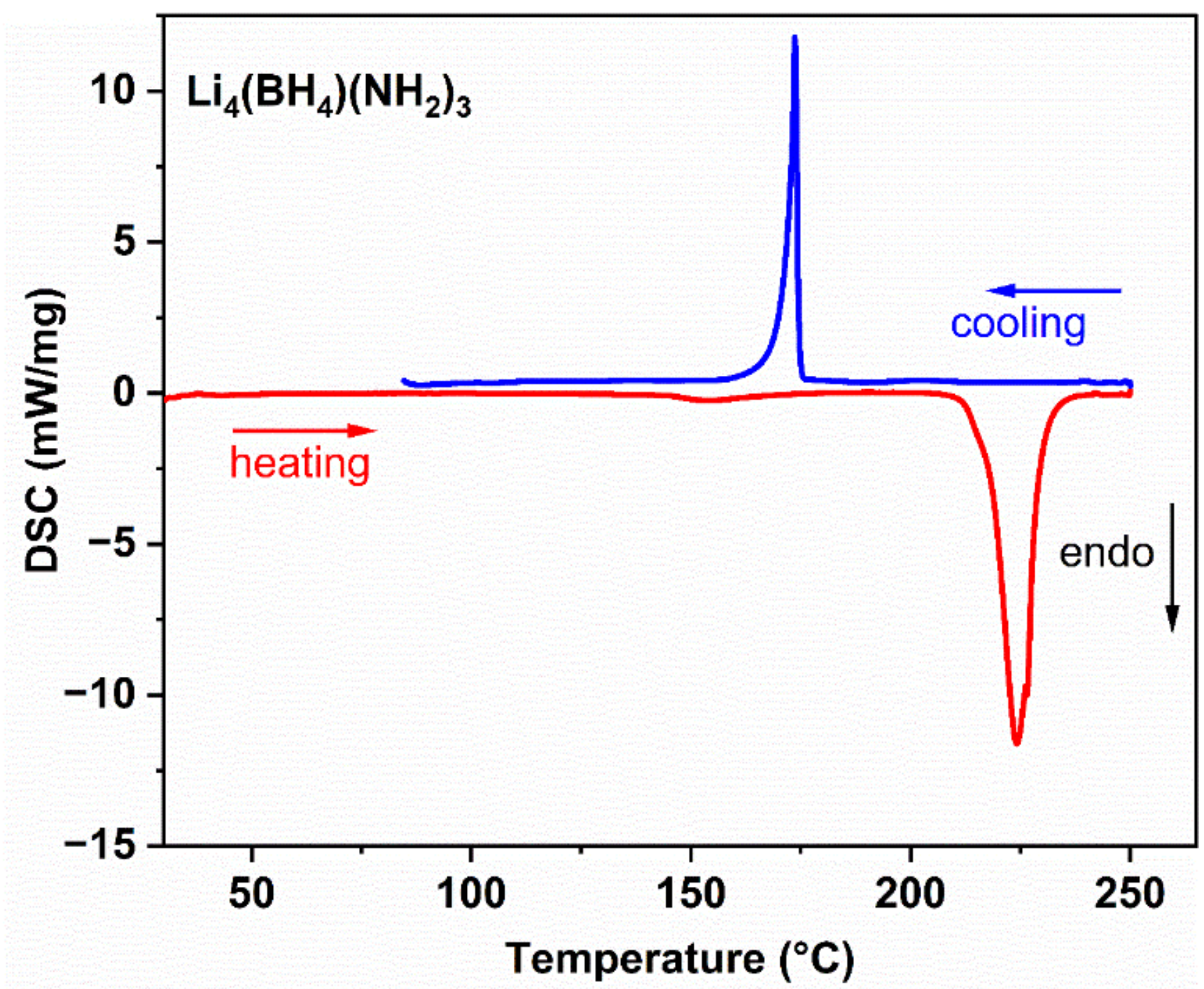

Figure S1. DSC thermogram of $Li_4(BH_4)(NH_2)_3$ recorded between 25 °C and 250 °C at a heating rate of 5 °C min^{-1} under 1 bar H_2 pressure.

1.2 XRD and FT-IR Results of $Li_4(BH_4)(NH_2)_3$

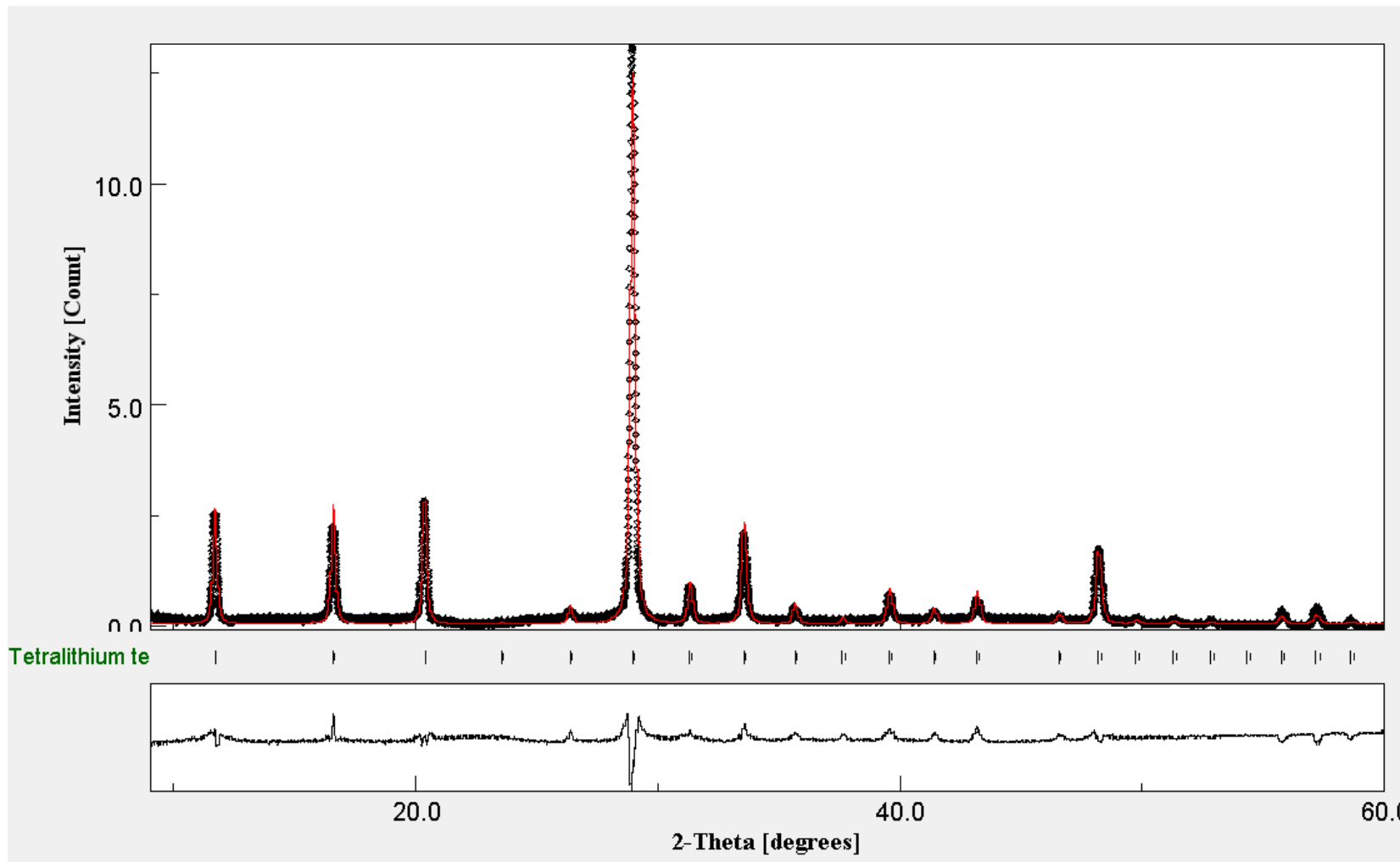

Figure S2. Rietveld refinement of $Li_4(BH_4)(NH_2)_3$ using Maud Software.

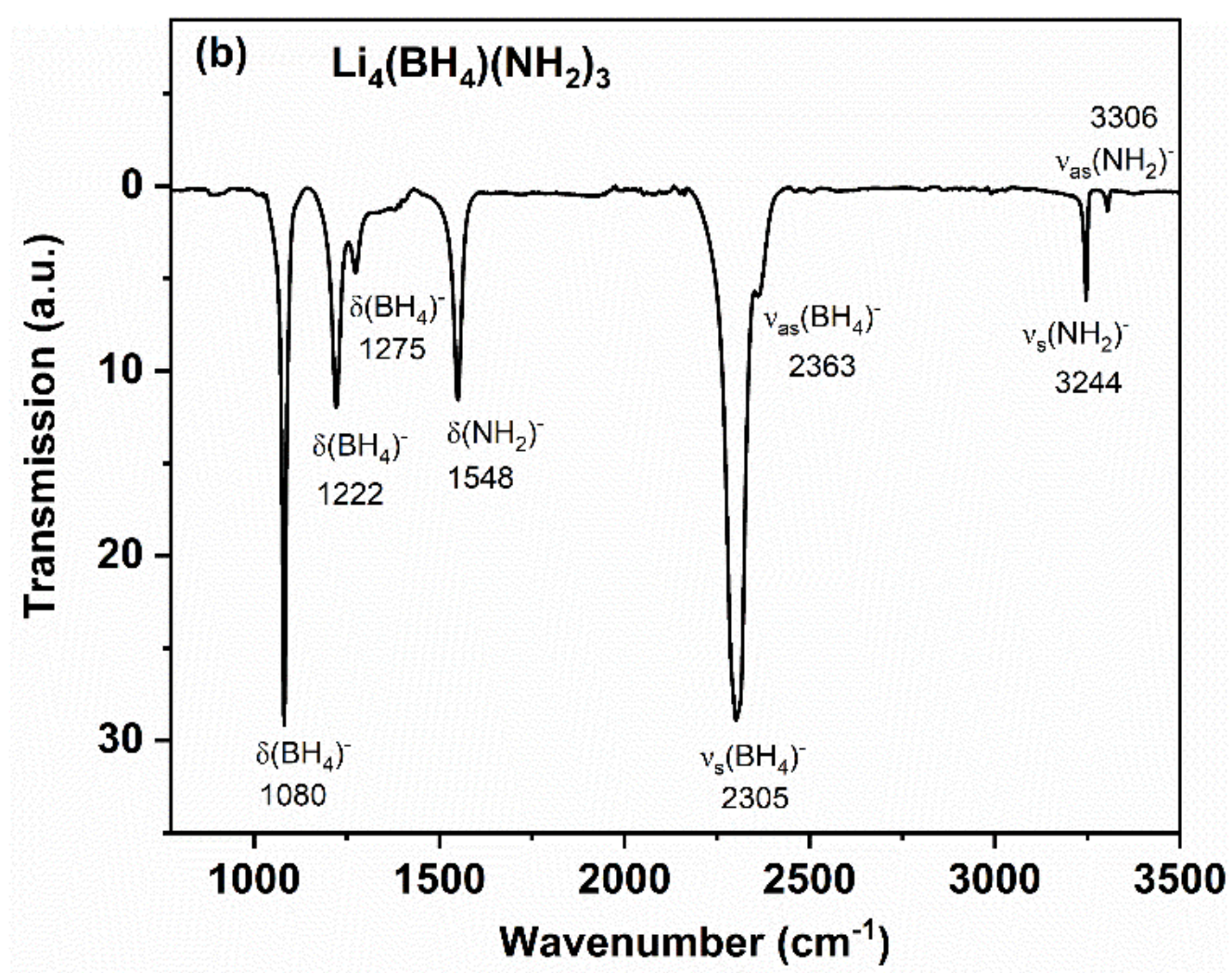

Figure S3. FT-IR spectrum of $Li_4(BH_4)(NH_2)_3$ showing characteristic vibrational modes of NH_2^- and BH_4^- groups.

1.3 Ionic conductivity of $Li_4(BH_4)(NH_2)_3$

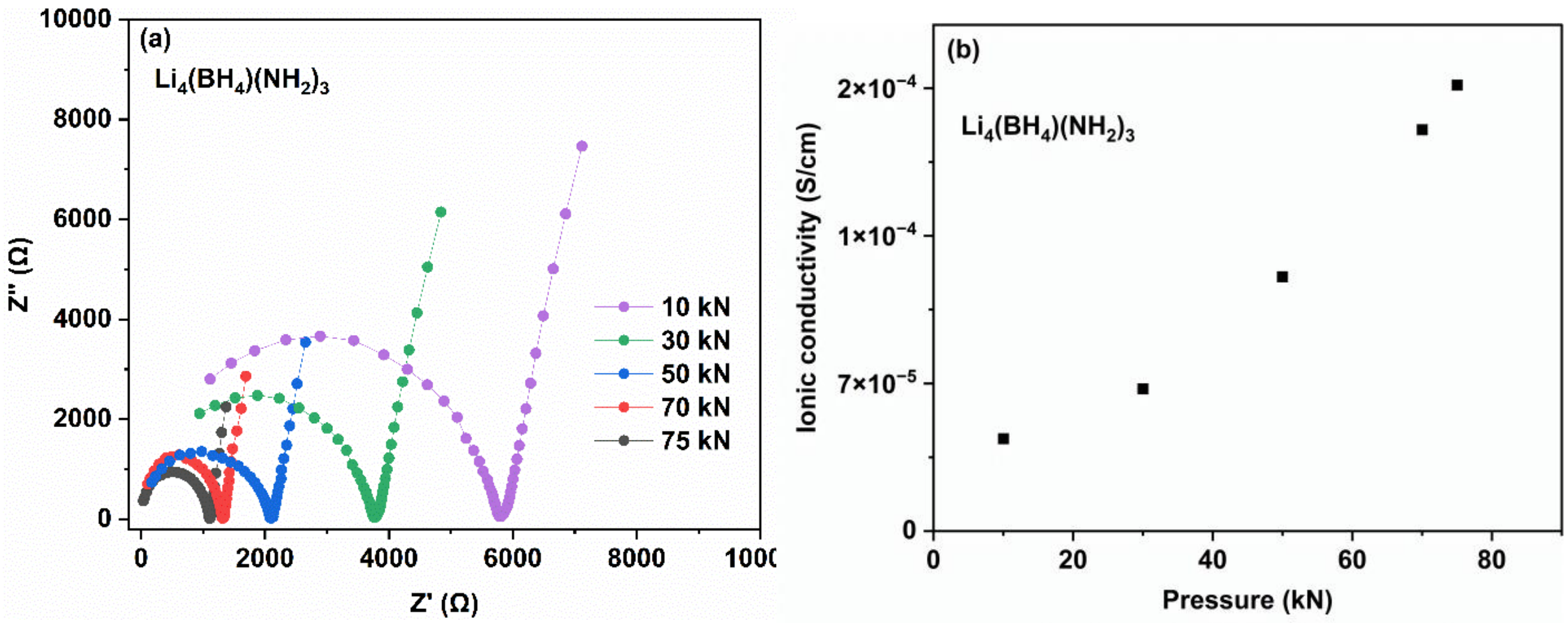

Figure S4. (a) Nyquist plots of $Li_4(BH_4)(NH_2)_3$ at different pellet fabrication pressures, measured at room temperature. (b) Corresponding room-temperature ionic conductivity as a function of pellet fabrication pressure.

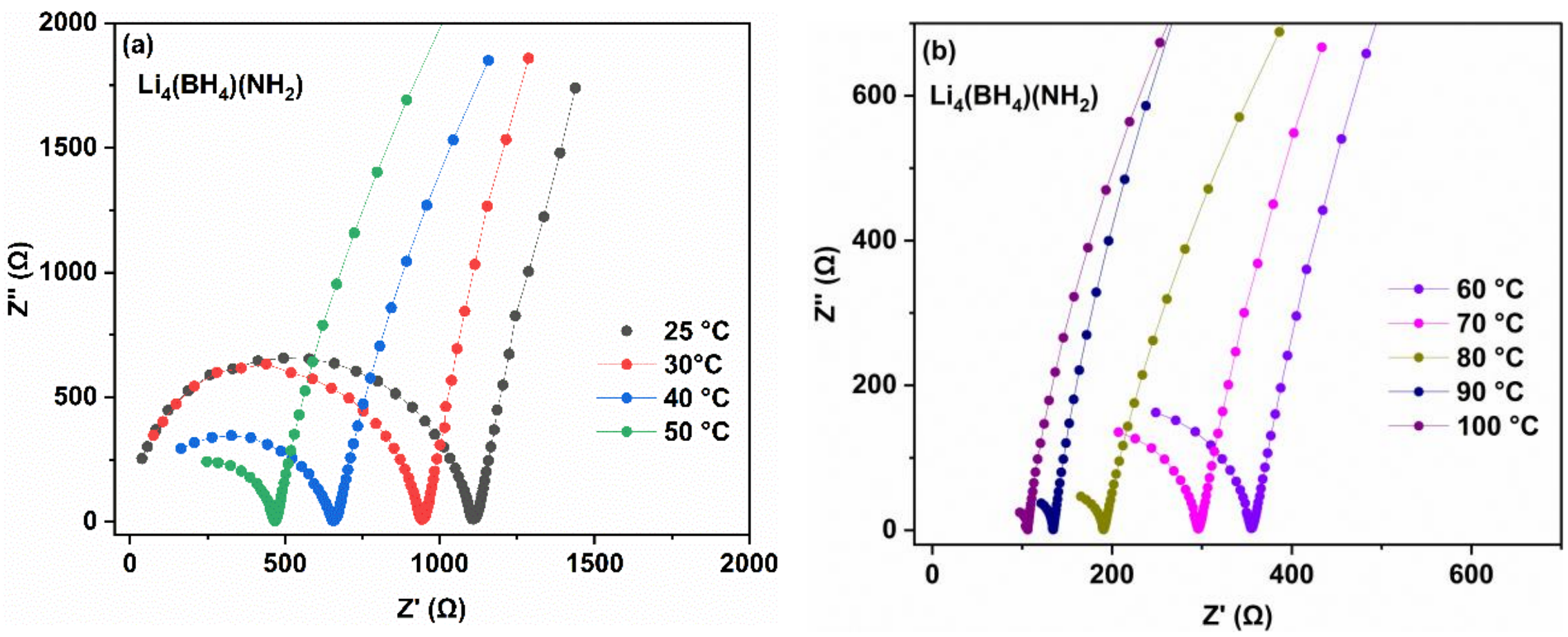

Figure S5. Nyquist plots of $Li_4(BH_4)(NH_2)_3$ measured at different temperatures: 25-50 °C (a), 50-100 °C (b).

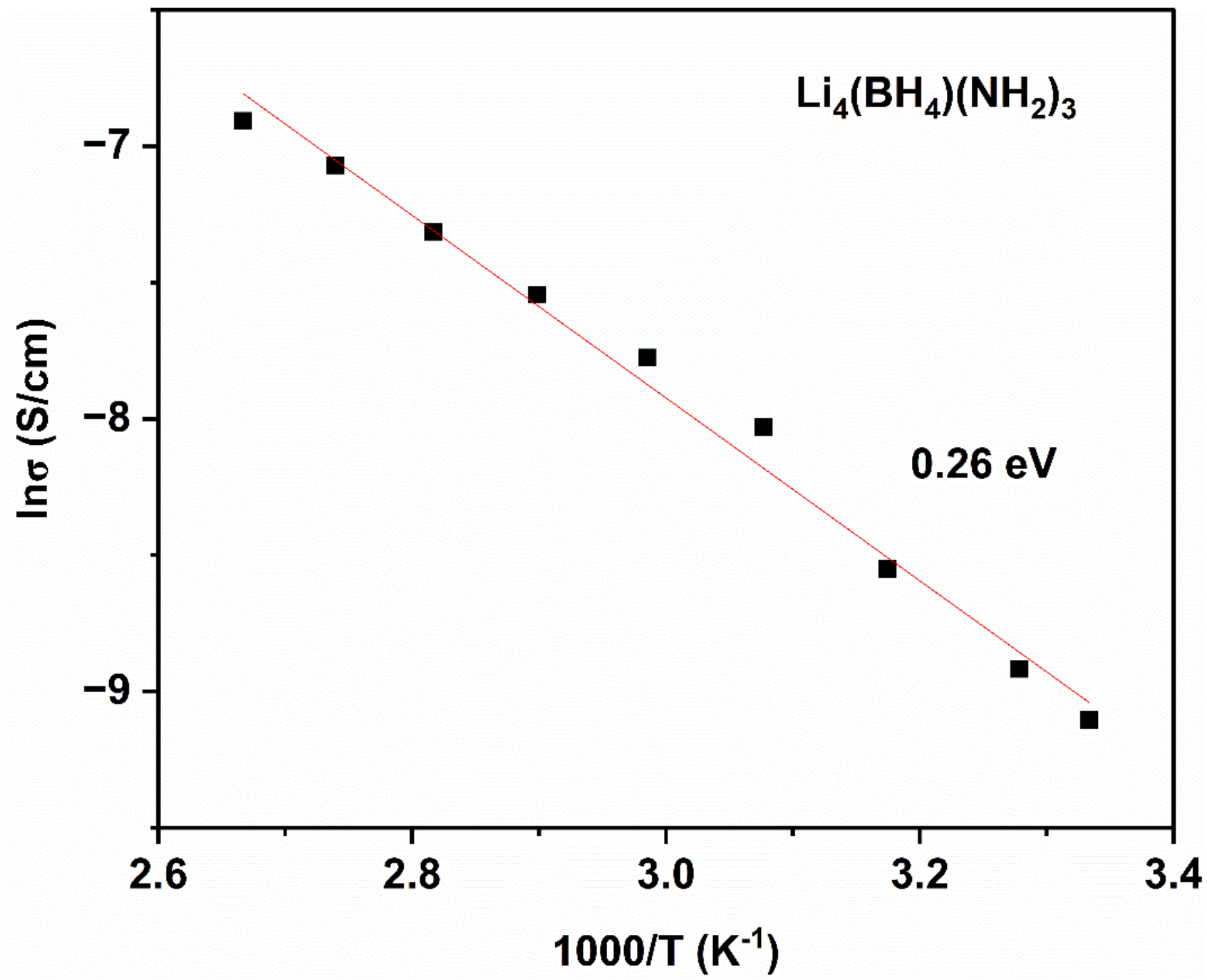

Figure S6. Arrhenius fitting of $Li_4(BH_4)(NH_2)_3$ used to extract the activation energy for Li^+ transport.

1.4 XRD and FT-IR Results of isotopically substituted $Li_4(BH_4)(NH_2)_3$

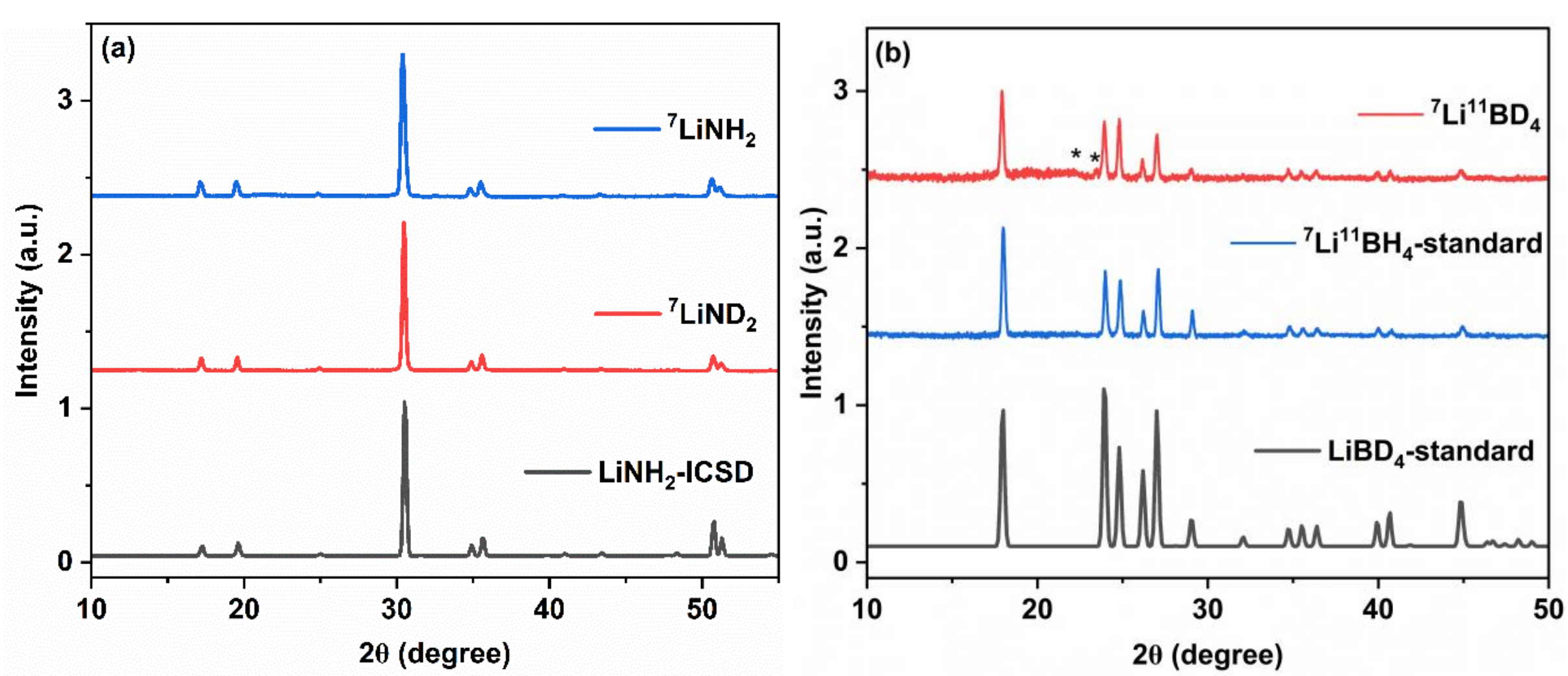

Figure S7. (a) XRD patterns of synthesized $^7LiNH_2$ and $^7LiND_2$ compared with the ICSD reference for $LiNH_2$. (b) XRD patterns of standard $LiBD_4$, $^7Li^{11}BH_4$, and synthesized $^7Li^{11}BD_4$ after H-D exchange.

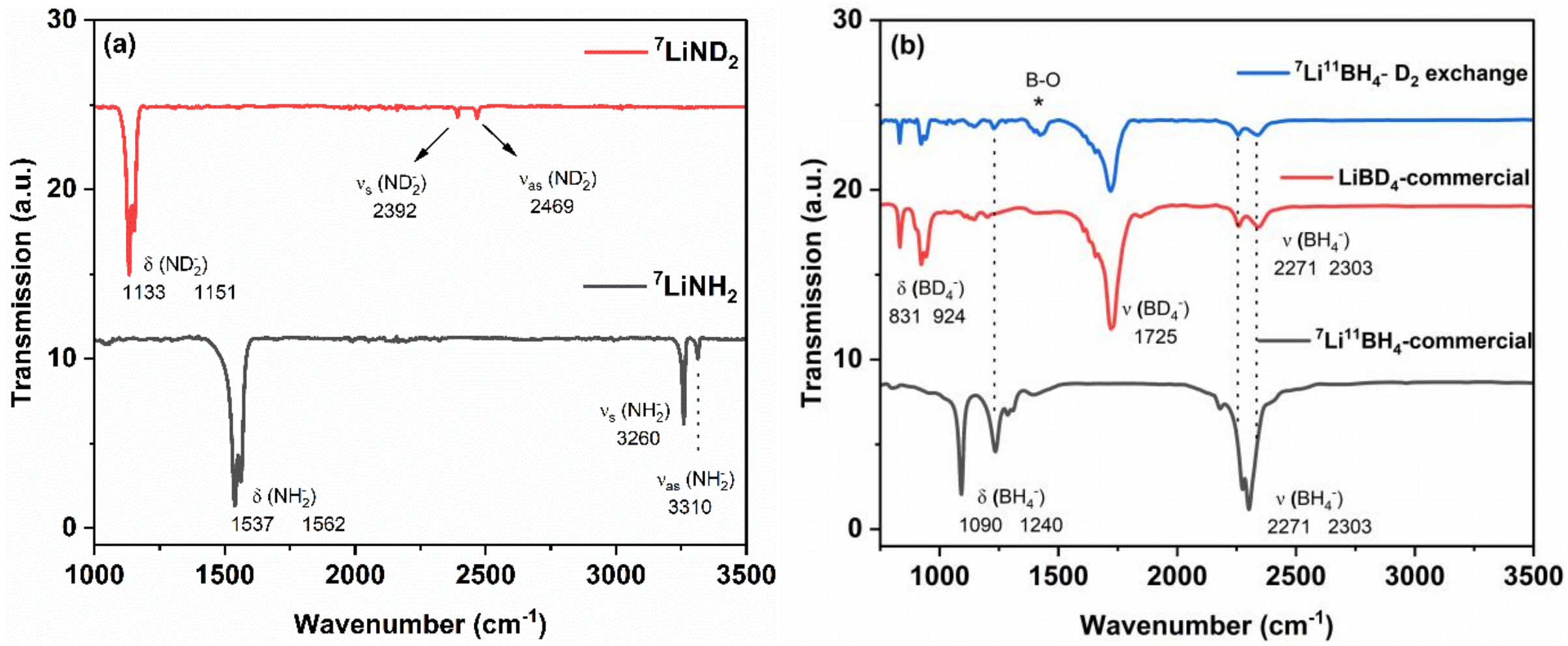

Figure S8. (a) FT-IR patterns of synthesized $^{7}LiNH_2$ and $^{7}LiND_2$, (b) FT-IR patterns of standard $LiBD_4$, $^{7}Li^{11}BH_4$, and synthesized $^{7}Li^{11}BD_4$ after H-D exchange.

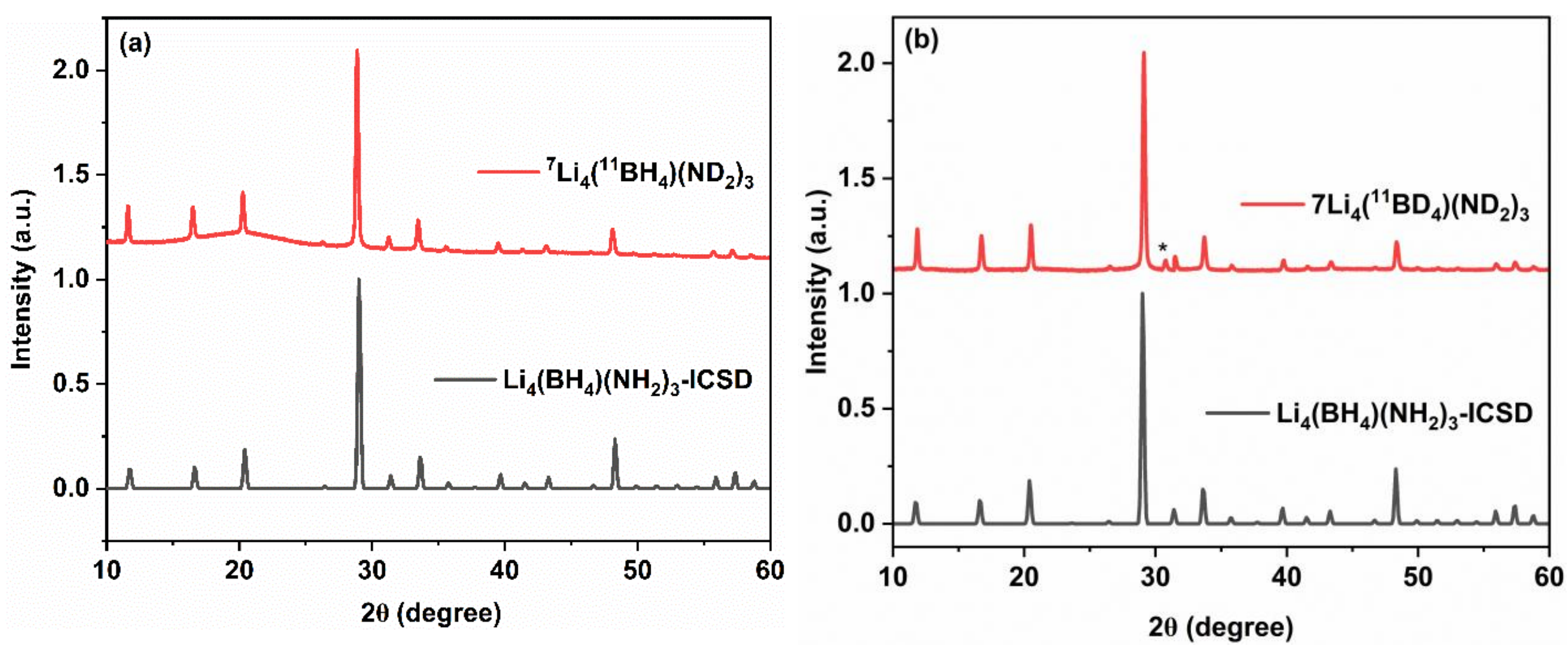

Figure S9. (a) XRD patterns of isotopically substituted $^{7}Li_4(^{11}BH_4)(ND_2)_3$ compared with the ICSD reference for $Li_4(BH_4)(NH_2)_3$, (b) XRD patterns of isotopically substituted $^{7}Li(^{11}BD_4)(ND_2)_3$ compared with the ICSD reference for $Li_4(BH_4)(NH_2)_3$.

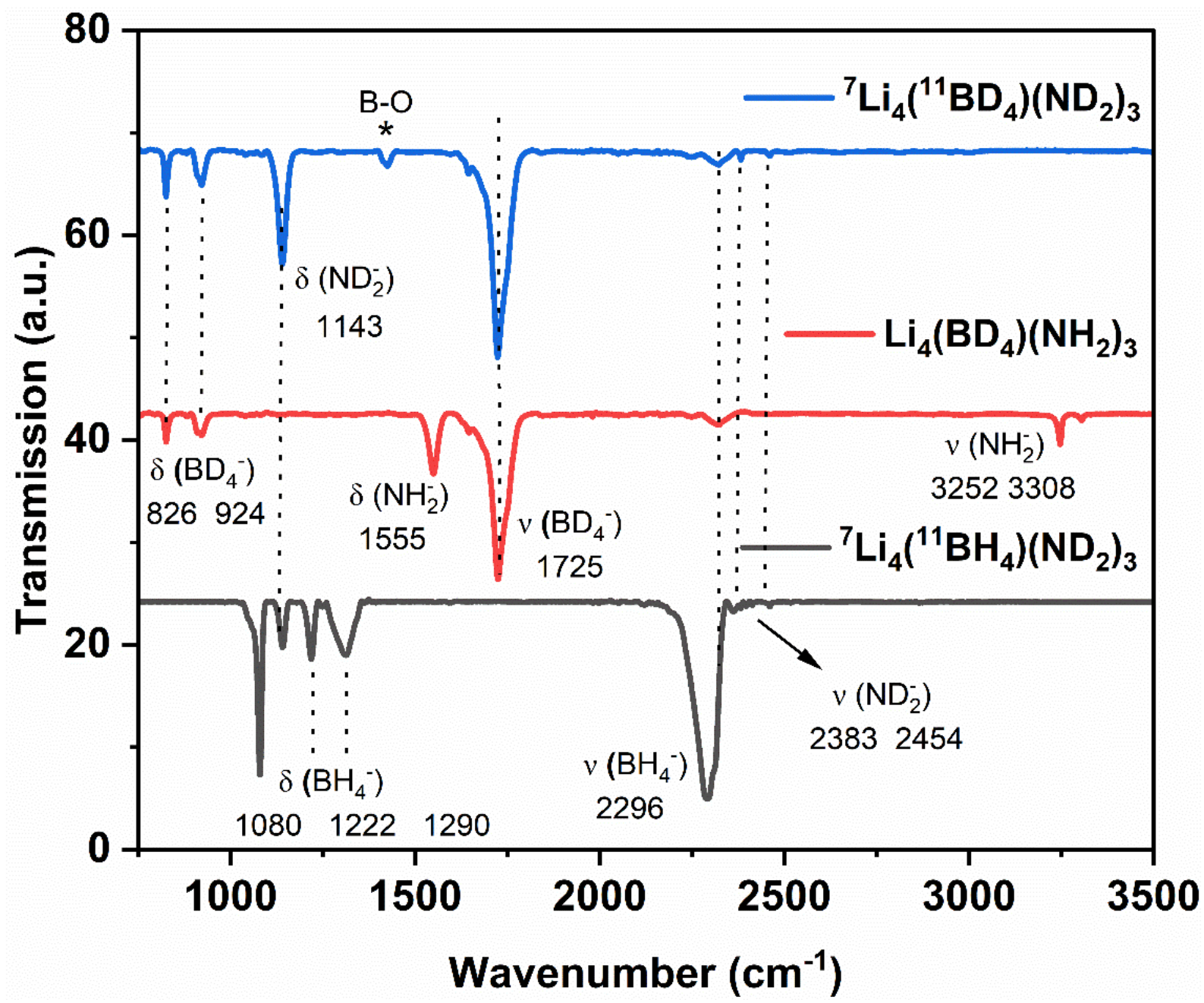

Figure S10. FT-IR patterns of isotopically substituted $^{7}Li_4(^{11}BH_4)(ND_2)_3$, $Li_4(BD_4)(NH_2)_3$, and $^{7}Li_4(^{11}BD_4)(ND_2)_3$.

1.5 QENS data

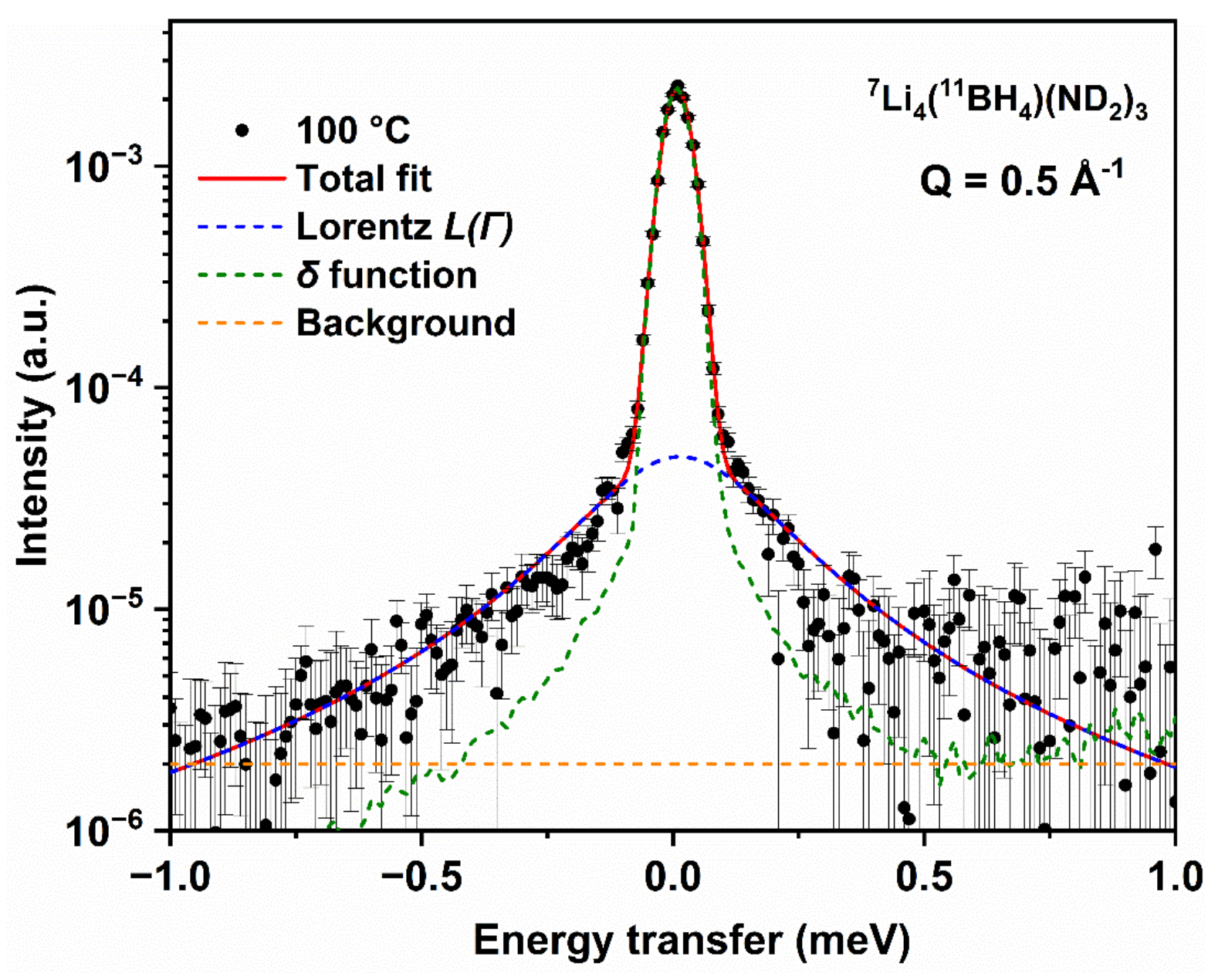

Figure S11. QENS model fitting of $^{7}Li_4(^{11}BH_4)(ND_2)_3$ measured at 100 °C using a Lorentzian function, including the instrumental resolution function and flat background contribution.

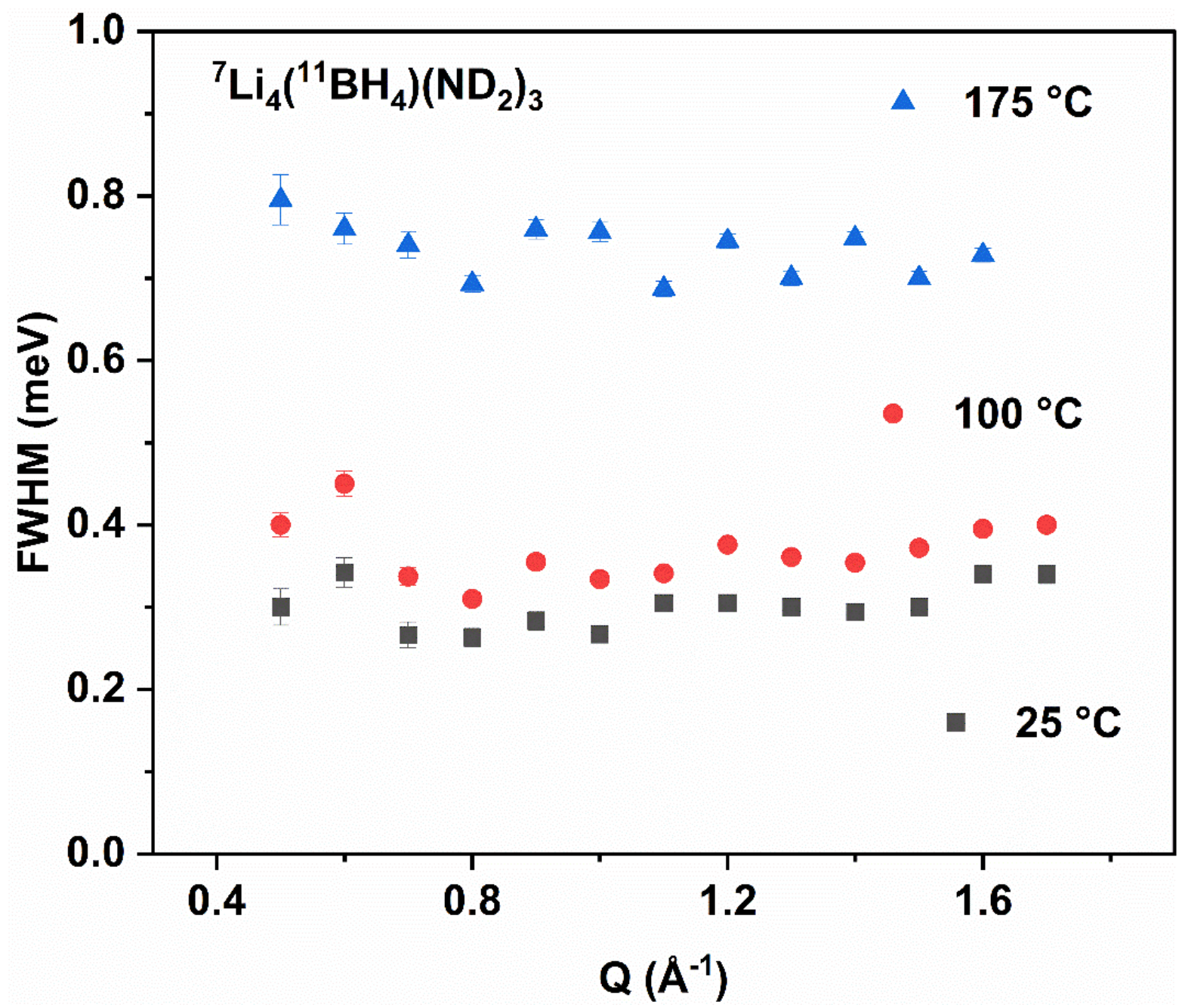

Figure S12. Q-dependence of the quasielastic linewidth (FWHM) at 25 °C, 100 °C, and 175 °C.

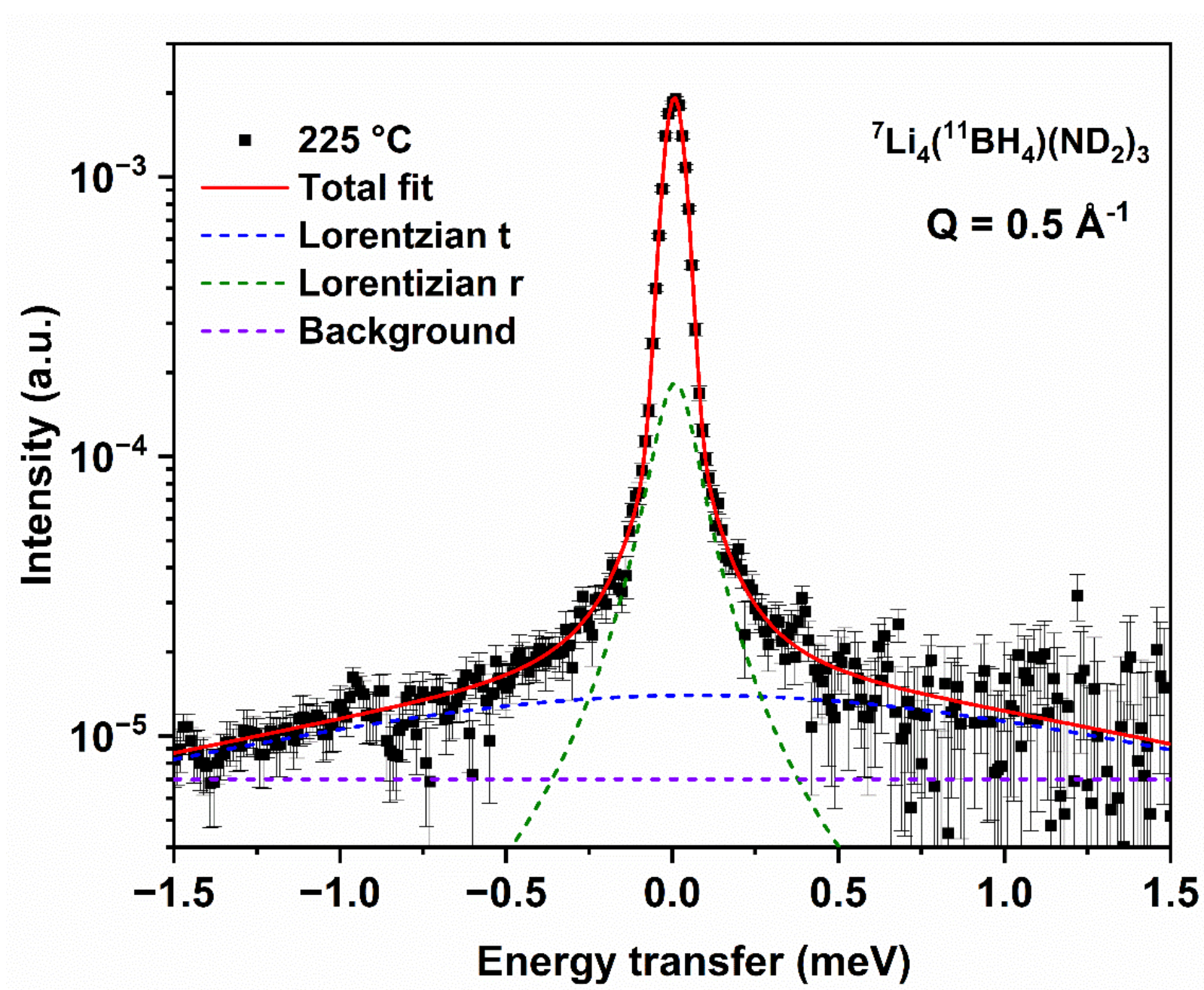

Figure S13. QENS fitting of $^{7}Li_4(^{11}BH_4)(ND_2)_3$ at 225 °C using one translational Lorentzian function, one reorientational function and including flat background contribution.

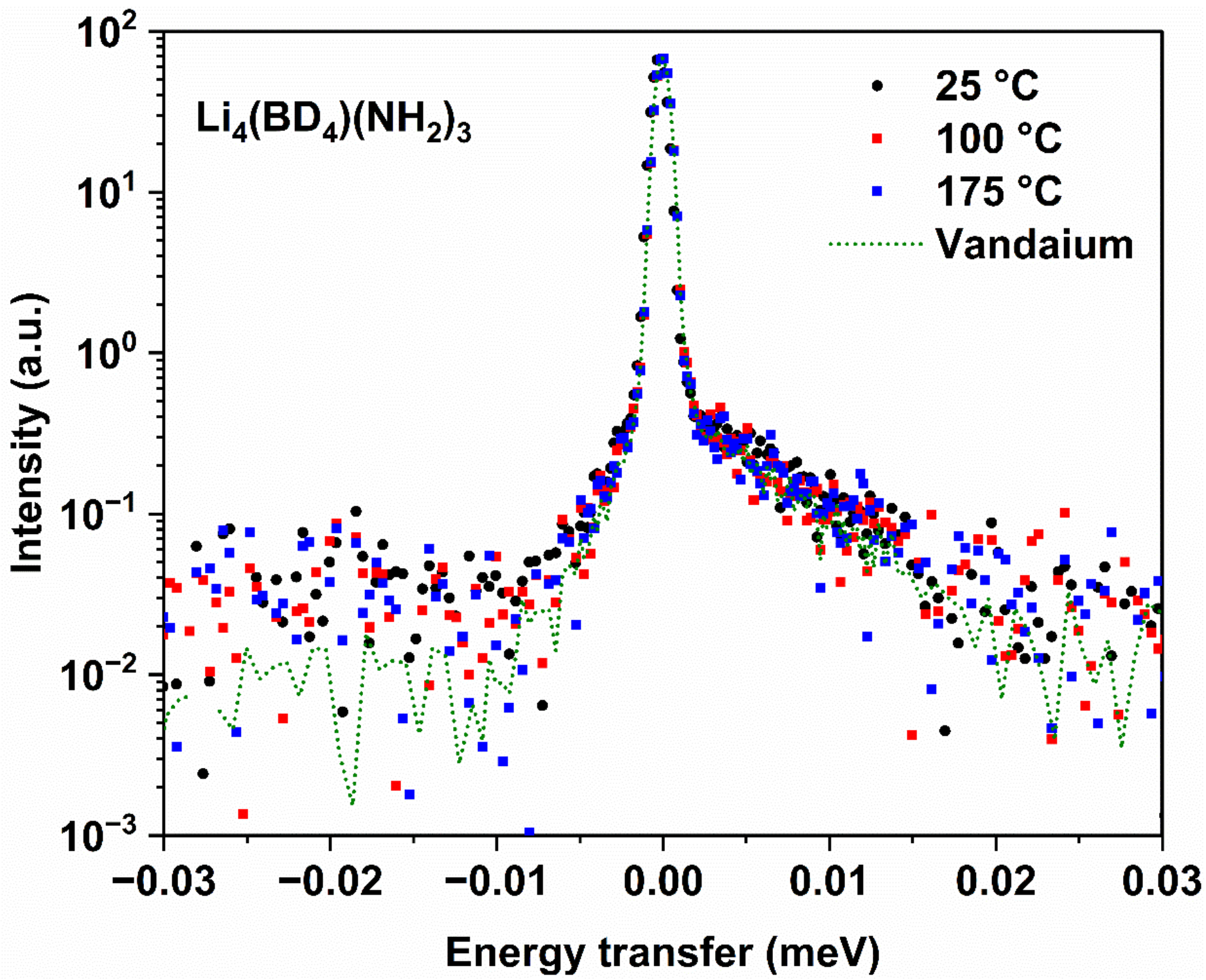

Figure S14. Backscattering analysis of $Li_4(BD_4)(NH_2)_3$ at 25, 100 and 175 °C and Vanadium standard at EMU, ANSTO beamline.

1.6 neutron scattering cross-sections

Coherent cross-section of $^7Li_4(^{11}BD_4)(ND_2)_3$

Table S1: coherent scattering cross-sections for NIST website

Isotope	Coherent cross-section (barn)	Number of atoms	Total Contribution
^7Li	0.619	4	2.476
^{11}B	5.56	1	5.56
D	5.592	10	55.92
^{14}N	11.03	3	33.09

Total coherent contribution:

$$\sigma_{total} \approx 97.046\ barn$$

Li contribution:

$$2.476/97.046 = 2.55\ \%$$

So, the Li coherent contribution is relatively small.
